# Canagliflozin, characterized as a HDAC6 inhibitor, inhibits gastric cancer metastasis

**DOI:** 10.3389/fonc.2022.1057455

**Published:** 2022-11-15

**Authors:** Dandan Jiang, Peizhi Ma

**Affiliations:** Department of Pharmacy, Henan Provincial People’s Hospital, People’s Hospital of Zhengzhou University, Zhengzhou, Henan, China

**Keywords:** gastric cancer, HDAC6, EMT, canagliflozin, enzyme inhibition

## Abstract

Gastric cancer is a common gastrointestinal cancer. Survival outcome for patients with the recurrence or metastasis remains poor due to the lack of effective targeting drugs. The mechanisms of non-histone acetylation modifications are key epigenetic regulations that participate in various biological processes. HDAC6 is mostly located in the cytoplasm to deacetylate non-histone substrates, which has been identified as a critical promoter of many oncogenic pathways in cancers, including gastric cancer. Nevertheless, its inhibitor has not been applied in gastric cancer clinically. In this study, we identified canagliflozin as an active HDAC6-targeted inhibitor from FDA-approved Drug Library by enzymatic assay. The strong affinity of the compounds with HDAC6 was further verified by surface plasmon resonance (SPR) and cellular thermal shift assay (CETSA). In addition, molecular docking showed that canagliflozin could bind to the active pocket of HDAC6 and form interactions with key residues. Further experiments revealed that canagliflozin could effectively inhibit the migration and epithelial-mesenchymal-transition (EMT) of gastric cancer cells *in vitro* and *in vivo*. These results reveal a novel finding that canagliflozin has the potential to be an effective agent in inhibiting gastric cancer metastasis.

## Introduction

Gastric cancer is one of the most common malignancies of the human digestive system in eastern Asia, with a high recurrence rate and a poor 5-year survival rate, especially among older males (1, 2). Most patients are diagnosed at an advanced stage (3, 4). The most lethal aspect of advanced gastric cancer is its ability to metastasize. For patients with metastatic gastric cancer, the currently accepted treatment options are still dominated by chemotherapeutic agents, which bring limited improvement in survival and significant side effects (5). Therefore, it is imperative and urgent to search for more efficient therapeutic strategies.

Increasing study revealed that mechanisms associated with non-histone acetylation modifications is recognized as important epigenetic regulatory processes involved in key cellular processes related to physiology and disease, such as gene transcription, DNA damage repair, cell division, signal transduction, protein folding, autophagy and metabolism (6). HDAC6 is mostly located in the cytoplasm to deacetylate non-histone substrates, such as the cytoplasmic protein substrates α-tubulin, cortactin, and heat shock protein 90, and HDAC6 is highly correlated with tumor malignancy (7–10). Besides, HDAC6 is highly expressed in estrogen receptor-positive breast cancer cells and accelerates the metastatic process of breast cancer by reducing the acetylation modification of α-tubulin, enhancing microtubule activity, and promoting cell adhesion and migration (9, 11). In leukemia cells, HDAC6 inhibitors can inhibit the process of α-tubulin deacetylation by HDAC6, disrupting the microtubule dynamics system and ultimately inhibiting cancer cell migration (12). Furthermore, HDAC6 inhibition can block the process of proliferation and invasion of gastric cancer cells (13, 14). Thus, targeting HDAC6 could be a promising therapeutic option for gastric cancer treatment.

Canagliflozin, produced from phlorizin, has anti-type 2 diabetes effects by inhibiting sodium/glucose co-transporter 2 (15, 16). Meanwhile, canagliflozin may have beneficial effects on some cardiovascular outcomes in patients with type 2 diabetes (17). Furthermore, accumulating evidence indicates that canagliflozin can regulate WNT/β-catenin or AMP-activated protein kinase/mammalian target of rapamycin (AMPK/mTOR) signaling, so that to block the growth of hepatocellular carcinoma and breast cancer (18–20). But the effect of canagliflozin on gastric cancer has not been identified.

Enzymes are common targets in high-throughput screening (21). Additionally, the repurposing of existing drugs already approved by the Food and Drug Administration (FDA) for human therapy is regarded as an effective strategy for drug development (22). In this study, we took advantage of FDA-approved drug library and indicated that canagliflozin effectively inhibits the enzymatic activity of HDAC6 and shows an excellent affinity with HDAC6. Canagliflozin dramatically suppressed the metastatic potential of gastric cancer cells by reversing the expression of EMT markers. Collectively, our data revealed that canagliflozin inhibits gastric cancer cell migration and EMT through targeting HDAC6 *in vitro* and *in vivo*.

## Materials and methods

### Enzymatic assay

FDA-approved Drug Library was purchased from APExBIO. The HDAC6 fluorescence assay was used to perform preliminary *in vitro* enzymatic activity tests on all compounds (23). Firstly, HDAC6 substrate Ac-Lys-AMC (Chinapeptides, China), human recombinant protein HDAC6 (100 ng/mL) (BPS bioscience, USA), and the compounds were incubated to be tested at 37°C in assay buffer containing 25 mM HEPES, pH 7.5, 100 mM NaCl, 2.5 mM KCl, 0.005% Tween-20, 1 mM MgCl_2_ and 0.1 mg/mL bovine serum albumin for 1 hour. Then, trypsin was added to liberate the fluorescent fragment. The fluorescence was measured by an automatic enzyme label analyzer (355/460 nm). The inhibition rate was calculated based on the fluorescence intensity measured at different concentrations of the compound to be tested. IC_50_ was calculated and fitted using GraphPad Prism 8.0.

### Surface plasmon resonance

SPR method of Biacore T200 (GE Healthcare, USA) was used to measure the binding affinity. HDAC6 was diluted in sodium acetate solution (pH 4.5) to a final concentration of 100 μg/mL. Then it was immobilized on a CM5 sensor chip (GE Healthcare, USA) by the amine coupling principle to achieve a target density of approximately 10,000 resonance units (RU). The immobilized HDAC6 was used to capture the compound to be measured. The running buffer contained 10 mM sodium phosphate (pH 7.5), 200 mM sodium chloride, 0.005% Tween-20, and 1% DMSO. The compounds to be measured were diluted in a running buffer at a maximum concentration of 5 μM. Data were recorded at 25°C. KD value was calculated by Biacore Evaluation Software.

### Molecular docking

A sketch of the test compound was drawn using ChemDraw 2014 and imported into MOE 2019. 3D structures of the compounds used for docking were then built using MOE 2019, and energy minimization was performed with a gradient of 0.01. X-ray crystal structure of HDAC6 (PDB: 6PYE) (24), obtained from Protein Data Bank with a good resolution of 1.48 Å, was prepared using MOE 2019 QuickPrep module. The co-crystal ligand of this protein was used to define the docked active site. GBVI/WSA dG was used as a scoring method to select the best pose for the test compound.

### Cell culture

Human gastric cancer cell line MGC-803 and HGC-27 were obtained from the National Collection of Authenticated Cell Cultures at Shanghai, China, and cells were maintained in Dulbecco’s modified of eagle’s medium (DEME) ((Biological Industries, Israel) with 10% fetal bovine serum (Biological Industries, Israel). Cells were cultured in a 37 °C incubator with 95% air and 5% CO_2_.

### Cellular thermal shift assay

The target engagement of canagliflozin in cells was confirmed by cellular thermal shift assay (CETSA) (25). After the human gastric cancer cell line, MGC-803 was seeded in the dish; the cells were incubated for another 12 h. Then, the compound was added to the medium with DMSO as a vehicle (final solvent concentration of 0.1% (v/v)) for 3 h. After that, cells were collected and resuspended in the PBS solution with heating at the indicated temperature. Then, the soluble fraction from the heat-treated cells was collected by centrifuge for 20 minutes at 12000 rpm, 4 °C. The supernatant was transferred to a new tube for the following western blot analysis.

### Wound healing assay

An artificial gap is generated on a confluent cell monolayer, and movement is tracked *via* microscopy or other imaging. Cells were seeded in multiple-well plates and incubated for 24 h. After cells reached 90% confluence, the wound was scratched using a 10 μL micropipette tip. Then, the medium was discarded, and cells were washed with PBS buffer once. After that, media containing the compound with indicated concentration was added into each well. Then, images were acquired before and after the 24 h incubation.

### Transwell assay

1 × 10^4^ cells in cell culture medium with 1% fetal bovine serum (Biological Industries, Israel) were seeded into the upper chamber of the transwell plate (Corning, USA). Meanwhile, 600 µl medium with 20% fetal bovine serum as the chemoattractant was added to the lower chamber of the transwell plate above. After indicated treatment and an additional 24 h incubation, the upper chamber was removed, and cells from the upper chamber were removed and wiped away with a cotton swab. Cells on the other side of the lumen were fixed in methanol and stained with 0.05% crystal violet staining solution. Finally, cells were imaged using a light microscope (Ts100, Nikon, Japan) and analyzed with Image J 1.47 (National Institutes of Health, USA).

### Western blot

After indicated treatment, cells were collected, and recommended amount of radio immunoprecipitation assay (RIPA) buffer (KMT Bio, China) was added to lysate the cells according to the instruction. Then, the concentration of the protein was quantified by bicinchoninic acid (BCA) based protein quantification kit (KMT Bio, China). After denaturing in the presence of 6×loading buffer (KMT Bio, China), the sample with the same number of samples were subjected to sodium dodecyl sulfate–polyacrylamide gel electrophoresis, and the protein to be tested was then transferred to the nitrocellulose membrane (Pall, USA) using wet transfer method. The membrane was then incubated with 5% non-fat milk at 37 °C for 1 h followed by an overnight incubation with the recommended concentration of antibody at 4 °C overnight. Afterwards, the membranes were washed three times with PBST containing PBS buffer and 0.1% Tween-20 for 10 min each. The secondary antibody was then diluted and incubated with the membrane at room temperature for 1 h. Finally, the membranes were washed another three times with PBST for 10 min each, and imaged with ECL chemiluminescence detection kit (ThermoFisher, USA).

### Lung metastasis

Six-week-old male BALB/c nude mice were purchased from SJA laboratory animals in Hunan, China. All mice were acclimatized to living conditions in a pathogen-free environment for one week prior to the experiment. 1 × 10^6^ stable transfections of MGC-803 cells with firefly luciferase (fLuc-MGC-803 cells) were injected into the tail vein of the mice. Mice were given 0, 25, or 50 mg/kg of the compound, separately, by gavage daily. 0, 5 weeks after i.v. injection, bioluminescence (BLI) signal of the fLuc-MGC-803 cells in mice were detected after intraperitoneal injection of substrate D-luciferin (150 mg/kg) by an IVIS Spectrum system. All mice were housed in specific pathogen-free conditions, and the protocol was approved by the Ethics Committee of the Zhengzhou University Health Science Centre.

### Statistical analysis

Data were analyzed using GraphPad Prism 8.0 (USA). *t*-test or one-way analysis of variance (ANOVA) was used for the statistical significance evaluation.

## Result and discussions

### Novel HDAC6 inhibitors selection

**Figure 1:**
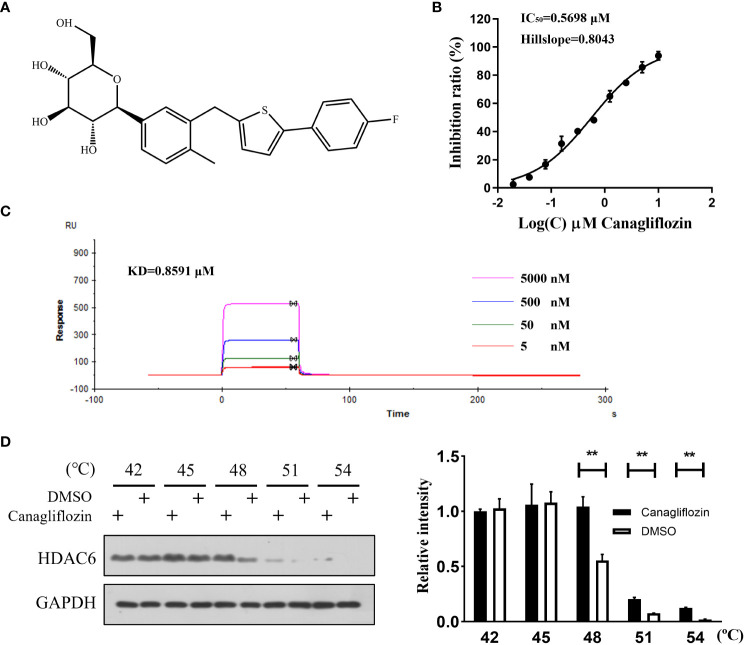
Canagliflozin binds to HDAC6 and inhibits its activity. **(A)** The structure of canagliflozin; **(B)** The HDAC6 inhibition curve of canagliflozin; **(C)** Dose-dependent binding curve of HDAC6 with canagliflozin at 5, 0.5, 0.05, 0.005 μM; **(D)** Stability of HDAC6 when MGC-803 cells were treated with or without canagliflozin at 42, 45, 48, 51 and 54 °C. Data were acquired with three repeats and presented as mean ± standard deviation. **P < 0.01.

An FDA-approved drug library was employed to screen novel HDAC6 inhibitors. For the primary screen, HDAC6 activity was calculated after incubation with the drugs in the concentration of 5 μM or vehicle (DMSO) using the enzymatic assay. The top twenty compounds without reported HDAC6 inhibitory activity were selected to evaluate IC_50_. Among them, canagliflozin (**Figure 1A**) showed the most potent inhibition with an IC_50_ of 0.5698 μM (**Figure 1B**). To deeply discuss the effect of canagliflozin, SPR was conducted to investigate the affinity between HDAC6 and canagliflozin. **Figure 1C** shows canagliflozin bound to HDAC6 with high affinity (KD = 0.8591 μM). To further confirm the target engagement, cellular thermal shift assay was applied to MGC-803 cells treated with canagliflozin or not. As shown in **Figure 1D**, canagliflozin can significantly stabilize HDAC6 at 48°C, 51°C, and 54 °C, respectively, suggesting that canagliflozin can bind to HDAC6 at cellular level.

### Intermolecular interactions of canagliflozin with HDAC6 by molecular docking

**Figure 2:**
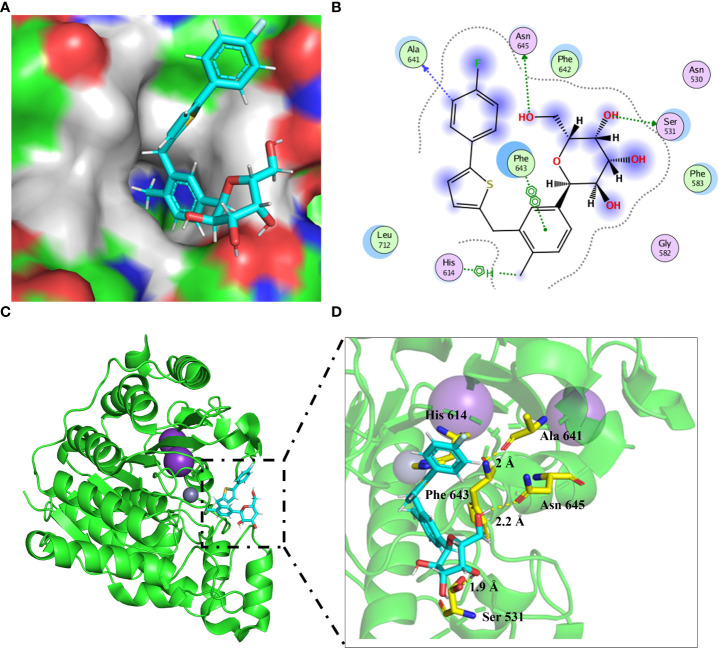
Molecular docking predicts the binding model between HDAC6 (PDB: 6PYE) and canagliflozin. **(A)** The binding pocket around the canagliflozin (cyan) was shown; **(B)** Two-dimensional diagram showing HDAC6 in combination with canagliflozin; **(C, D)** The canagliflozin (cyan) binding mode was shown in the cartoon model.

To further reveal the potential binding mechanism of canagliflozin to HDAC6, molecular docking was applied to predict the binding model. As shown in **Figure 2A**, canagliflozin occupied the hydrophobic pocket of HDAC6. Canagliflozin formed hydrogen bonds with Ser 531 (1.9 Å), Ala 641 (2 Å), and Asn 645 (2.2 Å) and π-πinteraction with Phe 643 (**Figures 2B–D**). Molecular docking furtherly proved that canagliflozin may bind directly to HDAC6 with high affinity.

### Canagliflozin exerts anti-migration activity *in vitro*

**Figure 3:**
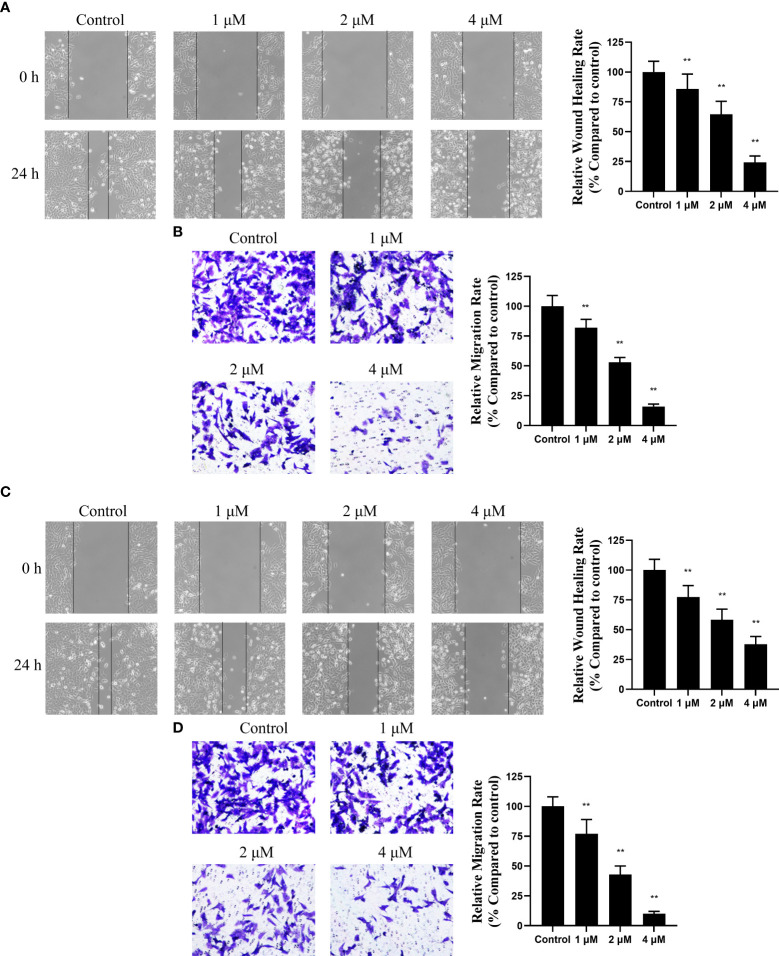
Canagliflozin can impair the migration of gastric cancer cell line MGC-803 and HGC-27. **(A)** Wound healing assay for the inhibitory activity evaluation of canagliflozin against MGC-803 cells for 24 h treatment; **(B)** Transwell assay for the inhibitory activity evaluation of canagliflozin against MGC-803 cells for 24 h treatment. **(C)** Wound healing assay for the inhibitory activity evaluation of canagliflozin against HGC-27 cells for 24 h treatment; **(D)** Transwell assay for the inhibitory activity evaluation of canagliflozin against HGC-27 cells for 24 h treatment. Data were acquired with three repeats and presented as mean ± standard deviation. **P < 0.01.

As HDAC6 was reported to promote EMT-mediated cell migration (26, 27), whether canagliflozin can inhibit cell migration and EMT remains to be verified. So, a wound healing assay was applied to MGC-803 cells for the preliminary assessment of the anti-migration activity of canagliflozin. Firstly, the cell viability assay was carried out. Canagliflozin can sightly inhibit MGC-803 cells with an IC_50_ of 12.29 ± 1.03 μM and HGC-27 cells with an IC_50_ of 14.61 ± 1.16 μM (**Figure S1**). Nevertheless, as shown in **Figure 3A**, canagliflozin can inhibit the wound healing of MGC-803 cells at low concentrations when cells were exposed to the compound for 24 hours. Meanwhile, the transwell experiment was also applied to semi-quantify the anti-migration ability of canagliflozin against MGC-803 cells. As indicated in **Figure 3B**, canagliflozin can dose dependently inhibit the migration of MGC-803 cells significantly. To further exclude the cell specific effect of canagliflozin, wound hound healing assay and transwell experiment were also performed in the other gastric cancer cell line HGC-27, and results suggested that canagliflozin can inhibit the wound healing (**Figure 3C**) and cell migration ability (**Figure 3D**) of HGC-27 cells in a dose dependent manner. All these results confirmed that canagliflozin can significantly inhibit cell migration in cells.

### Canagliflozin blocks EMT process

**Figure 4:**
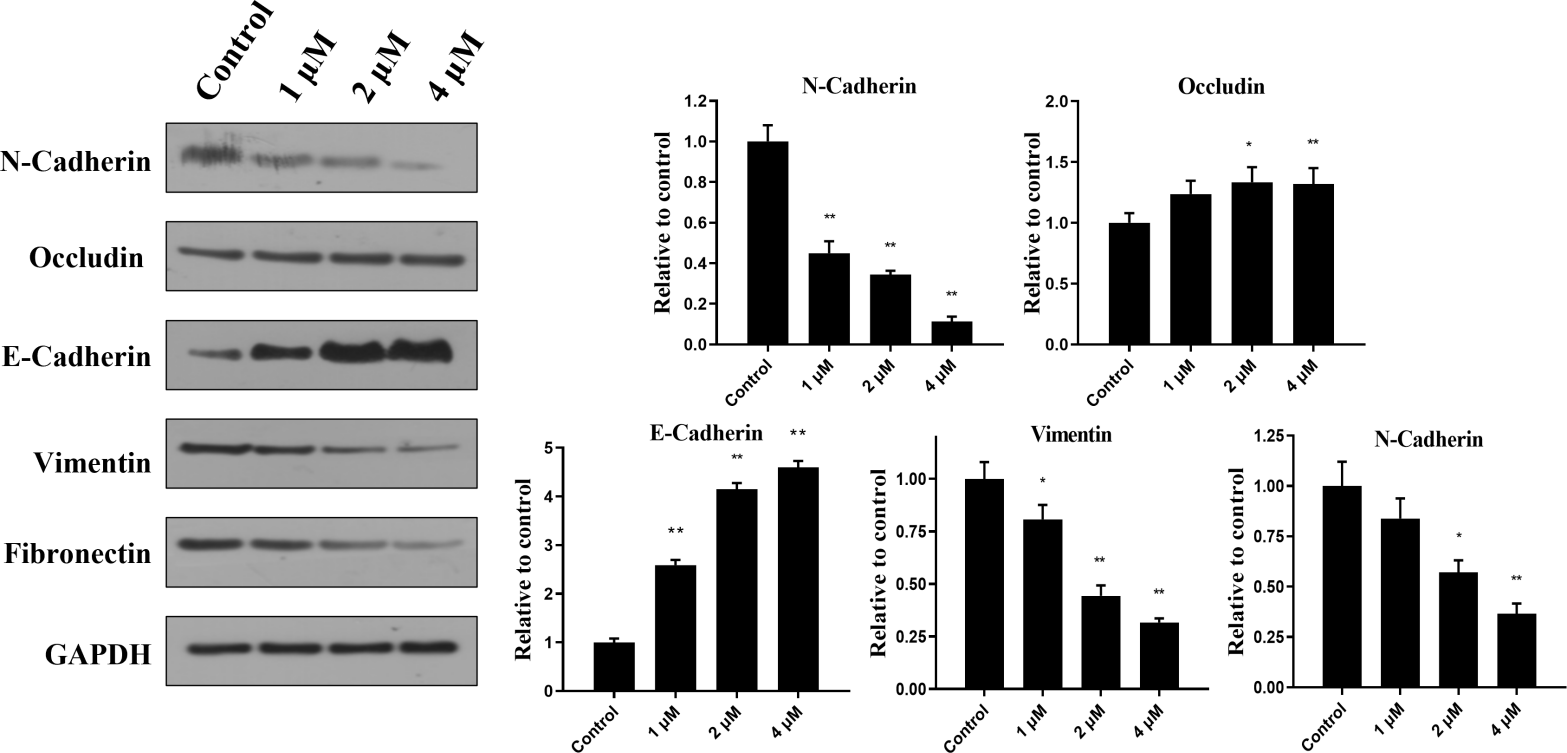
Canagliflozin can inhibit the EMT of MGC-803 cells for 24 h treatment. Expressions of EMT markers were evaluated after cells were treated for 24 h. Data were acquired with three repeats and presented as mean ± standard deviation. *P < 0.05; **P < 0.01.

Activation of EMT is a key process in cancer cell metastasis, in which epithelial cells acquire mesenchymal cell characteristics and increase cell motility and migration capacity. Several studies have reported that GC patients are associated with EMT activation (28, 29). To further reveal the mechanism that may contribute to the migration inhibitory effect of canagliflozin, markers of EMT in MGC-803 cells treated with canagliflozin were examined. As suggested in **Figure 4**, canagliflozin can dose dependently induce the expressions of the epithelial cell markers Occluding and E-Cadherin, while the expressions of mesenchymal cell markers N-Cadherin, Vimentin, and Fibronectin were significantly decreased when cells were exposed to canagliflozin for 24 h. Consistent with the migration related results as above, these data suggested that canagliflozin reversed EMT of MGC-803 cells.

### Canagliflozin exerts anti-migration activity *in vivo*

**Figure 5:**
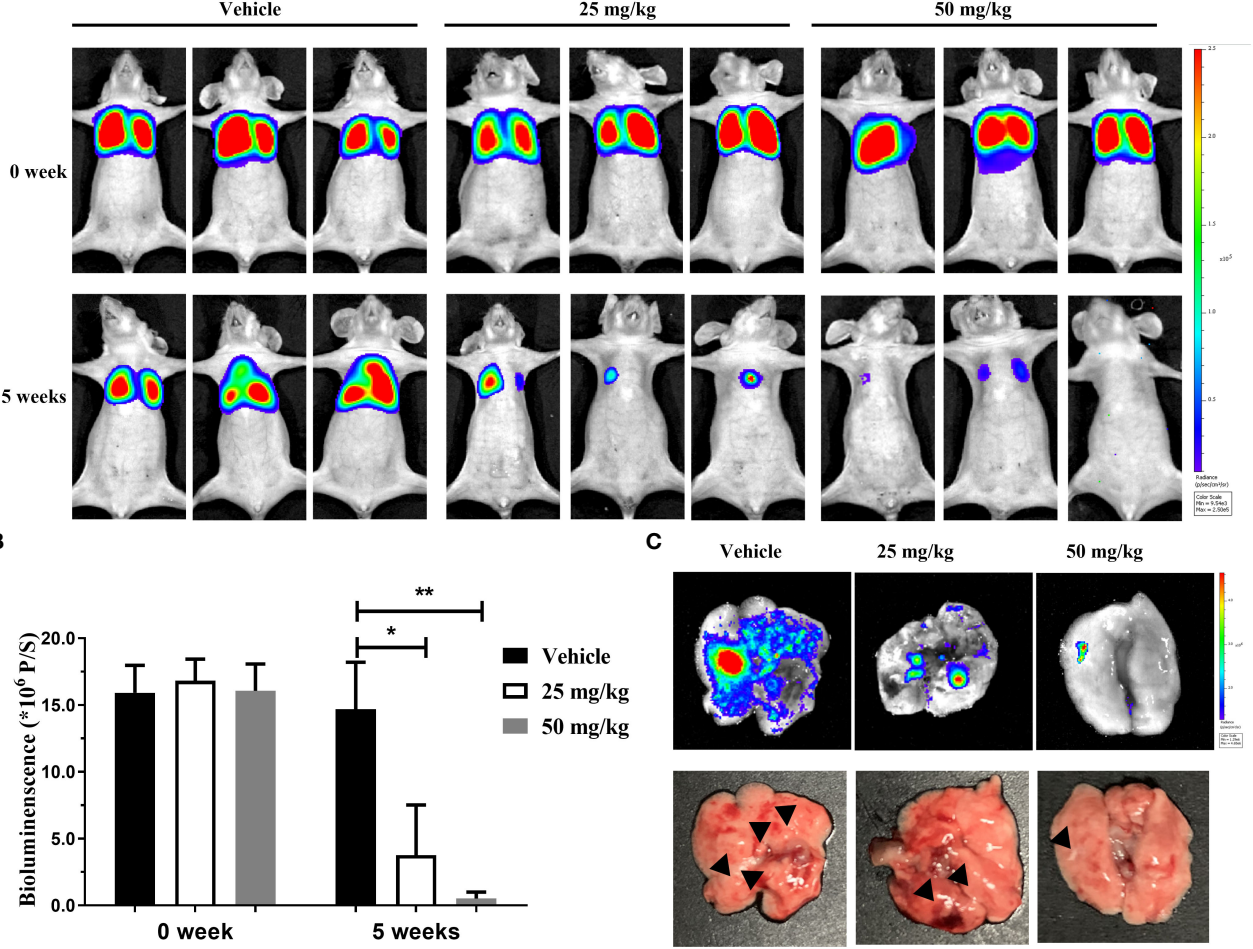
Canagliflozin can inhibit the lung metastasis of MGC-803 cells *in vivo.* **(A)** Representative serial bioluminescence images of mice with intravenous injection of fLuc-MGC-803 cells; **(B)** Total photon flux of BLI signal (representative of total tumor burden) in mice; **(C)** BLI images and morphological changes in lungs. Data were presented as mean ± standard deviation. *P < 0.05; **P < 0.01.

To further confirm the anti-migration ability of canagliflozin *in vivo*, MGC-803 cell line stably expressing luciferase (fLuc-MGC-803 cell line) was constructed. The lung metastasis model constructed by tail vein injection of 1× 10^6^ fLuc-MGC-803 cells was used to detect the anti-metastasis activity of canagliflozin *in vivo*. Results demonstrate that canagliflozin effectively inhibits MGC-803 metastasis at 25 mg/kg and 50 mg/kg compared to vehicle (**Figures 5A** **, B**). In addition, the group treated with canagliflozin resulted in a lower number of metastatic nodules than the control group (**Figure 5C**). These data suggest that canagliflozin has potential as an anti-metastatic agent *in vivo*.

## Conclusion

As gastric cancer is one of the leading causes of cancer-related death worldwide, it is urgent to discover new agent for gastric cancer treatment, and HDAC6 is the target we chose for the application of drug repurposing strategy in this study due to the significant role of HDAC6 in the progression of gastric cancer. Herein, we identified canagliflozin as a potent HDAC6 inhibitor with IC_50_ value of 0.5698 μM and KD value of 0.8591 μM. At the cellular level, canagliflozin can stabilize HDAC6, indicating that canagliflozin bound HDAC6 at the cellular level. EMT is an important mechanism for migration and wound healing that can promote tumor progression (27). HDAC6 inhibition is proved to upregulate E-cadherin, a marker of EMT, making breast cancer cells or peritoneal mesothelial cells less susceptible to undergoing EMT (12). HDAC6 abrogation also reversed TGF-β-mediated EMT in breast cancer cells (12). The same phenomenon was also revealed in esophageal squamous cell carcinoma by disrupting chaperone function of HSP90 (30). Besides, HDAC6 can also activate the phosphorylation of signal transducer and activator of transcription 3 on Tyr-705 and upregulate ZEB1 in MDA-MB-231 breast cancer cells to enhance EMT (9). We also demonstrated that canagliflozin can blocks the process of EMT and cell migration through the inactivation of HDAC6 in MGC-803 and HGC-27 gastric cancer cells *in vitro*. Correspondingly, canagliflozin interrupt the formation of lung metastases *in vivo*.

There are three HDAC6 selective inhibitors in clinical trials for cancer treatment, including ACT-1215, ACT-124, and KA2507 (31). Similar to these three HDAC6 selective inhibitors, canagliflozin showed good enzyme inhibition activity and dissociation constants to nanomolar concentrations. It is worth noting that compared to these known HDAC6 selective inhibitors, canagliflozin exhibits a widely disparate skeletal structure, which can complement and extend the discovery of new inhibitors. Although more and more studies reveal that HDAC6 may serve as an oncogene in gastric cancer, few HDAC6 inhibitors are selected for gastric cancer treatment, especially for metastasis (32). Otherwise, as a drug proved by FDA, canagliflozin will be ability to be rapidly developed and applied, compared to other HDAC6 selective inhibitors in preclinical trials. Refer to the HDAC6 selective inhibitors in clinical trials for solid tumor treatment, the extension experiment of canagliflozin may be carried out on metastatic or advanced gastric cancer by using alone or in combination with immunotherapy such as PD-1/PD-L1 antibody Nivolumab or chemotherapy such as Paclitaxel to explore the application of canagliflozin (33, 34).

All in all, canagliflozin was identified to target HDAC6 and served as a potent HDAC6 inhibitor, and it may be developed as potential therapeutics for gastric cancer.

## Data availability statement

The original contributions presented in the study are included in the article/**Supplementary Material**. Further inquiries can be directed to the corresponding author.

## Ethics statement

The animal study was reviewed and approved by Zhengzhou university.

## Author contributions

DJ: conceptualization, data curation, methodology, and software, writing – original draft. PM: data curation, validation, and writing – review and editing. All authors contributed to the article and approved the submitted version.

## Funding

This work were funded by the National Natural Science Foundation of China (Grant No. 82003682), Provincial Key R&D and Promotion Special (Scientific Problem Tackling) Project of Henan Province (Grant No. 212102311033) and Medical Science and Technology Program of Henan Province (Joint construction project, Grant No. LHGJ20200026).

## Conflict of interest

The authors declare that the research was conducted in the absence of any commercial or financial relationships that could be construed as a potential conflict of interest.

## Publisher’s note

All claims expressed in this article are solely those of the authors and do not necessarily represent those of their affiliated organizations, or those of the publisher, the editors and the reviewers. Any product that may be evaluated in this article, or claim that may be made by its manufacturer, is not guaranteed or endorsed by the publisher.
