# Supplementary material for: Canagliflozin, characterized as a HDAC6 inhibitor, inhibits gastric cancer metastasis
Source: Front Oncol. 2022 Nov 15;12:1057455. doi: 10.3389/fonc.2022.1057455 (PMC9705739; doi:10.3389/fonc.2022.1057455)
Supplement: Supplementary file 1 [file Image_1.pdf]

Supplementary Material

Cell Viability Assay

MGC-803 cells and HGC-27 cells were seeded with an initial density of 4000 cells per well in the 96-well plate. Then plates were incubated in a 37 °C incubator containing 5% CO_2 for 24 h. Canagliflozin was added to the medium from 128 μM by 1:1 ratio, and dimethyl sulphoxide (DMSO) was added as the control. After 24 h coincubation, CCK-8 (10 μL) was added to each well and incubated in a 37 °C incubator for 3 h. The absorbance was obtained with a multimode plate reader (PerkinElmer Envision, USA) at 450 nm and IC_{50} was calculated by GraphPad Prism 8.0.

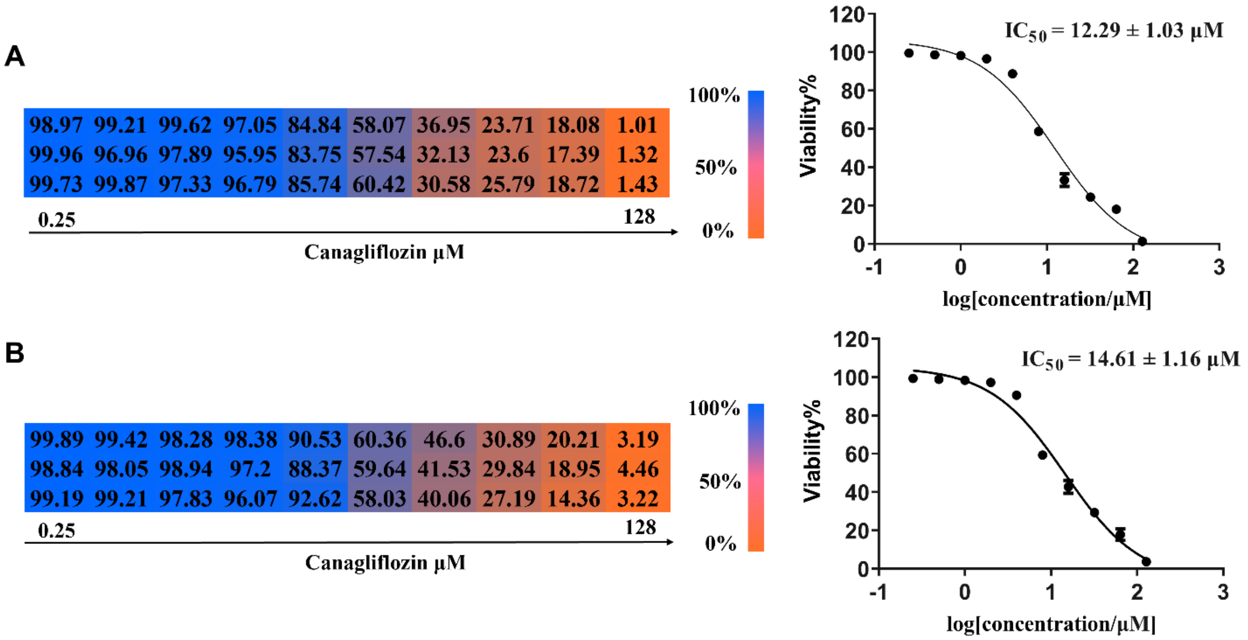

Supplementary Fig. S1. The inhibition effercts of the canagliflozin on (A) MGC-803; (B) HGC-27 gastric cancer cell lines. Data were aquired with three repeats and presented as mean ± standard deviation.
